# Efficacy of methimazole before the administration of radioactive iodine in the management of Graves’ disease: a systematic review and meta-analysis

**DOI:** 10.1590/1516-3180.2022.0225.R1.19102022

**Published:** 2023-01-09

**Authors:** Ikeoluwapo Kendra Bolakale-Rufai, Imodoye Abioro, Samuel Osobuchi Ngene, Yohannes Woldeamanuel

**Affiliations:** I MD. Physician, Department of Medicine, University College Hospital Ibadan, Ibadan, Oyo, Nigeria.; II MD. Physician, Department of Medicine, University College Hospital Ibadan, Ibadan, Oyo, Nigeria.; III MPH. Postgraduate Scholar, Swansea University Medical School, Swansea University, Wales, United Kingdom.; IV MD, PhD. Expert Physician, Medical Scientist and Instructor at Department of Neurology, School of Medicine, Stanford University, California, United States.

**Keywords:** Methimazole, Graves disease, Elements, radioactive, Hyperthyroidism, Anti-thyroid drugs, Radioactive iodine, Hyperthyroidism secondary

## Abstract

**BACKGROUND:**

The efficacy of anti-thyroid drugs in conjunction with radioactive iodine therapy in the management of Graves’ disease is still controversial.

**OBJECTIVE:**

To compare the efficacy of pretreatment with methimazole before the administration of radioactive iodine for the treatment of Graves’ disease.

**DESIGN AND SETTING:**

A systematic review and meta-analysis was conducted at a teaching/tertiary hospital in Ibadan, Nigeria.

**METHODS:**

A systematic search of the PubMed, Embase, Cochrane Library, and Web of Science databases was performed from inception to December, 2021.

**RESULTS:**

Five studies with 297 participants were included. There was no difference in the risk of persistent hyperthyroidism when radioactive iodine was used in conjunction with methimazole compared with when radioactive iodine was used alone (relative risk: 1.02, 95% confidence interval, CI: 0.62–1.66; P = 0.95, I^2^ = 0%). Subgroup analysis based on the duration between discontinuation of methimazole and the administration of radioactive iodine showed a lower risk of persistent hyperthyroidism when methimazole was discontinued within 7 days before radioactive iodine use, although this did not reach statistical significance (risk ratio: 0.85, CI: 0.28–2.58).

**CONCLUSIONS:**

The use of methimazole before radioactive iodine administration was not associated with an increased risk of persistent hyperthyroidism. Concerns about medication toxicity and adverse effects should be considered when clinicians make decisions on combination therapies for the treatment of Graves’ disease.

**PROSPERO REGISTRATION:**

CRD42020150013, https://www.crd.york.ac.uk/prospero/display_record.php?RecordID=150013.

## INTRODUCTION

Graves’ disease is an immune system disorder that results in an unregulated and overproduction of thyroid hormones due to circulating antibodies in the blood.^1,2,3^ The antibodies produced bind to the thyrotropin receptor and activate glandular function, resulting in hyperthyroidism.^2^ Graves’ disease leads to major cardiovascular and psycho-cognitive complications if left untreated, thus contributing to significant morbidity and mortality.^3,4^

As a leading cause of hyperthyroidism worldwide with an incidence of 30 cases per 100,000 persons per year in the United States, it is imperative to understand the pathophysiology and treatment modalities for the management of Graves’ disease.^5,6,7^ According to the 2016 American Thyroid Association guidelines for the diagnosis and management of hyperthyroidism and other causes of thyrotoxicosis, patients with overt Graves’ hyperthyroidism should be treated with any of the following modalities: radioactive iodine therapy, anti-thyroid drugs, and thyroidectomy.^5^

In the United States, radioactive iodine therapy (RAI) has been the most preferred therapy by physicians, with 59.7% of clinical endocrinologists opting for this as the primary therapy for an uncomplicated case of Graves’ disease.^8,9^ There has also been an increasing trend toward the use of anti-thyroid drugs (ATD), as this is the preferred first-line treatment for Graves’ disease by thyroidologists in Europe, Latin America, and Japan.^5,10,11,12,13^ However, a network meta-analysis has suggested higher relapse rates with ATDs (52.7%) than with RAI (15%).^3^

Although there is widespread and accepted use of radioactive iodine and anti-thyroid drugs individually, there is no consensus regarding their use in conjunction.^14^ It has been noted in the literature that following radioiodine therapy, an acute rise in thyroid hormone levels could occur, thus triggering a clinical exacerbation of symptoms.^15,16,17^ It has also been postulated that anti-thyroid medications such as methimazole could have radioprotective attributes and are thus beneficial for patients receiving radioactive iodine therapy.^18,19^ While some authors have explored the use of adjunct anti-thyroid drugs before radioactive iodine therapy,^9,19,20,21,22,23,24,25,26^ others prefer the use of anti-thyroid medications continuously during radioactive iodine^14,27^ and after radioactive iodine treatment.^28,29^

The varying study designs (retrospective studies, narrative reviews), disease population (toxic multinodular goiter, toxic adenoma), interventions (use of carbimazole and propylthiouracil), and the interval between therapies (use of ATD before, during, or after RAI) in the established literature produce considerable heterogeneity, which makes it difficult to reach a conclusion on the efficacy of anti-thyroid drugs in conjunction with radioactive iodine therapy.

## OBJECTIVE

We conducted a systematic review and meta-analysis of randomized controlled trials to evaluate the efficacy of treatment with methimazole before the administration of radioactive iodine compared to the use of radioactive iodine therapy alone for the treatment of Graves’ disease.

## METHODS

### Search strategy

The PubMed, Embase, Cochrane Library, and Web of Science electronic databases were searched for randomized controlled trials comparing adjunctive anti-thyroid drug use with radioactive iodine therapy versus radioactive iodine only in the treatment of Graves’ disease, from inception to December, 2021. The search terms included “Hyperthyroidism,” “Radioactive iodine,” and “Antithyroid drugs”. There were no restrictions on language or publication period. The searches were rerun immediately before the final data extraction and analyses, with further studies retrieved for inclusion.

### Study identification and selection

**Figure 1.**
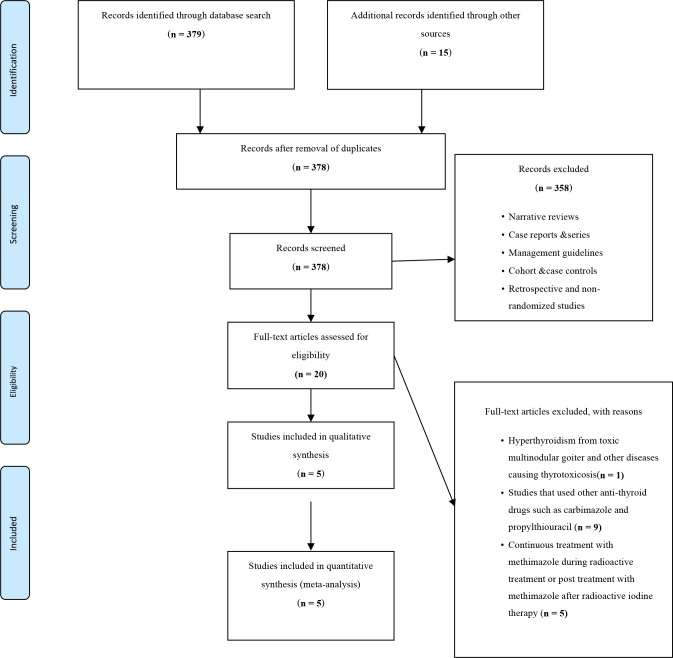
The Preferred Reporting Items for Systematic reviews and Meta-Analyses flow chart for study selection.

The Preferred Reporting Items for Systematic Reviews and Meta-Analyses^30^ was used as a guide for the identification and selection of studies (Figure 1).Two investigators independently screened and reviewed the titles and/or abstracts retrieved using the search strategy to identify titles that potentially met the inclusion criteria. The full texts of these potentially eligible studies were retrieved and independently assessed for eligibility by the two review team members. Disagreements between the two over the eligibility of the selected studies were resolved through consensus with a third reviewer.

Randomized clinical trials that compared adjunctive anti-thyroid medications with radioactive iodine therapy were deemed eligible for an in-depth review. Subsequently, 20 full-text articles were assessed for eligibility. Randomized clinical trials that evaluated initial treatment with methimazole before the administration of radioactive iodine therapy, regardless of the duration of treatment, were selected for final data extraction. We excluded studies that utilized anti-thyroid medications other than methimazole (such as carbimazole propylthiouracil) and those that administered methimazole either continuously or post-radioiodine therapy. We also excluded studies that incorporated other causes of hyperthyroidism, such as toxic multinodular goiter, because the focus was on Graves’ hyperthyroidism. This systematic review was specified in a registered protocol (PROSPERO: CRD42020150013, https://www.crd.york.ac.uk/prospero/display_record.php?RecordID=150013) before data extraction commenced.

### Risk of bias assessment

The risk of bias for studies incorporated in the systematic review and meta-analysis was assessed using the Cochrane risk of bias tool for randomized control trials. The studies were assessed for the following domains: random sequence generation, allocation concealment, blinding of participants and personnel, blinding of outcome assessment, incomplete outcome data, selective reporting, and other biases. The studies were further judged as ‘low risk,’ ‘some concerns, ’or‘ high risk.’

### Data extraction and synthesis

The RevMan 5.4 software (The Cochrane Collaboration, Oxford, United Kingdom)was used to perform the meta-analysis. The primary outcome measure was evidence of persistent hyperthyroidism after treatment (methimazole and radioiodine therapy in the experimental arm and radioiodine only in the control arm). The presence of hyperthyroidism after treatment was considered a “treatment failure.” Hypothyroid or normal thyroid values following treatment were stratified to be under the same class as “non-hyperthyroid state,” and thus considered a treatment success. Thyroid status was determined based on the clinical and laboratory criteria used for each clinical trial. The secondary outcome measure was the duration of discontinuation of adjunctive treatment with methimazole and its effect on the cure rates in patients with Graves’ disease.

We employed the random-effects meta-analysis model and inverse variance weighting method. A summary of the intervention effect for each study was provided by calculating the risk ratios and corresponding 95% confidence intervals (CI) for the main dichotomous variables: hyperthyroidism or non-hyperthyroidism. Heterogeneity was assessed using both the Q test and I-squared statistics. An I^2^ value greater than 50% was considered indicative of substantial heterogeneity. Forest plots were generated to evaluate the risk of publication bias.

## RESULTS

A total of 378 studies were identified through multiple database searches. Twenty full-text articles were assessed for eligibility, of which five randomized control trials were included in the final qualitative synthesis and meta-analysis. Full-text articles that were excluded were those with a patient population that had hyperthyroidism from other causes (n = 1), used other anti-thyroid medications (n = 9), and those with continuous treatment or treatment with methimazole after radioiodine (n = 5). Only trials that used methimazole as the drug of choice for the initial medical treatment were selected. The follow-up duration varied among the eligible studies; thus, studies were analyzed independently based on the duration of follow-up and evaluation of thyroid status at each visit. This was done to limit heterogeneity and incorporate all values into the final data synthesis. In the final meta-analysis, 289 patients who received methimazole before radioactive treatment were randomized to the treatment arm, while 335 were assigned to the control arm and received radioactive iodine therapy only.

### Study characteristics

The clinical trials included in this study were conducted in Brazil (n = 2), Slovenia (n = 1), and the United States of America (n = 2). All included studies were randomized trials, with the patient population being adults with Graves’ disease. The follow-up duration ranged from 14 days to one year (Table 1).

**Table 1.** Baseline characteristics of participants in included trials

Study	Country	AgeRAI+MMZ	AgeRAI	SexRAI+MMZ(M/F)	SexRAI(M/F)	No. assigned to RAI+MMZ	No. assigned to R AI	Duration of MMZ discontinuation (days)	FT4 RAI+MMZ(pmol/L)	FT4RAI(pmol/L)	Follow up(months)
Andrade et al.^20^	Brazil	37.6	34.5	2/25	4/24	23	28	4	61.78 ± 5.15	59.20 ± 5.92	1
Andrade et al.^22^	Brazil	37.4	35.1	2/27	4/28	29	32	4	59.20 ± 27.00	57.90 ± 21.90	12
Braga et al.^21^	United States	43.0	35.0	6/10	0/18	16	18	6	44.30 ± 21.00	66.80 ± 35.70	8
Burch et al.^23^	United States	42.0	36.0	7/14	2/19	21	21	6	80.00 ± 45.00	52.00 ± 40.00	0.5
Pirnat et al.^24^	Slovenia	43.5	46.8	8/42	8/51	50	59	7	20.40 ± 9.10	38.00 ± 17.80	1,3,6,&12

RAI = radioactive iodine; MMZ = methimazole; FT4 = free tyrosine.

**Figure 2.**
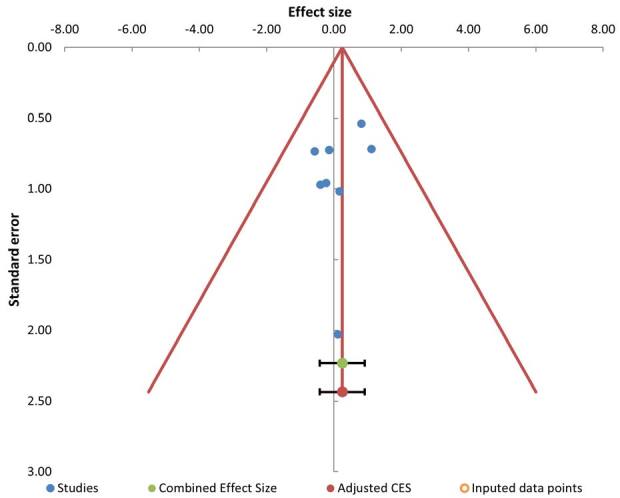
Funnel plot of publication bias in selected studies.

Minimal heterogeneity was found in the trials regarding the diagnostic criteria for hyperthyroidism. All studies utilized clinical assessments, suppressed thyroid stimulating hormone levels, thyroid hormone levels, 24-hour radioactive iodine uptake, and antibody levels to diagnose patients. Based on Cochrane’s tool to assess the risk of bias, there was no study with a high risk of bias (Table 2). One study had a low risk of bias, while others had concerns regarding the randomization process and selection of reported data (Figure 2).

**Table 2.** Risk of bias assessment of included studies

Unique ID	Experimental	Comparator	Randomization process	Deviations from intended interventions	Missing outcome data	Measurement of the outcome	Selection of the reported result	Overall
Andrade et al.^22^	RAI+MMZ	RAI	+	+	+	+	?	+
Braga et al.^21^	RAI+MMZ	RAI	?	+	+	?	?	?
Andrade et al.^20^	RAI+MMZ	RAI	+	+	+	+	?	?
Burch et al.^23^	RAI+MMZ	RAI	+	?	+	+	+	?
Pirnat et al.^24^	RAI+MMZ	RAI	?	?	+	?	?	?

+ = Low risk; ? = Some concerns. RAI = radioactive iodine; MMZ = methimazole.

### Outcome analysis

**Figure 3.**
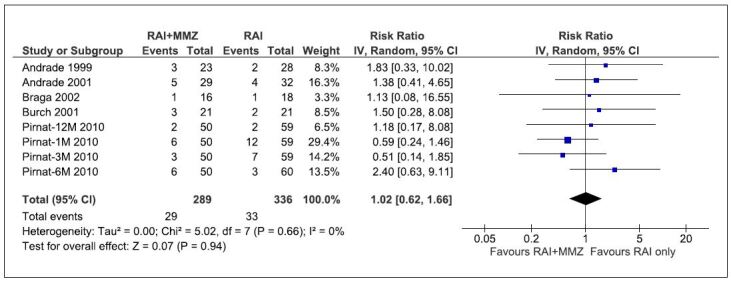
Forest plot of comparison: radioactive iodine + methimazole versus radioactive iodine only; outcome: persistent hyperthyroidism.

Using a random effects model for the meta-analysis, pretreatment with methimazole in conjunction with radioactive iodine therapy alone was not associated with an increased risk of persistent hyperthyroidism at follow-up in patients with Graves’ disease (relative risk, RR:1.02, 95% CI: 0.62–1.66; P = 0.95). Heterogeneity among the treatment effects was low (I2 = 0%). The funnel plot displayed an asymmetric distribution (Egger’s t-test = 1.31, P = 0.238) (Figure 3).

A subgroup was created based on the interval between the discontinuation of methimazole and radioactive iodine therapy. Subsequently, a subgroup analysis was performed, which revealed an RR of 1.52 (CI: 0.28–8.18) for studies with a 4-day duration of discontinuation of methimazole before radioactive therapy, while an RR of 1.38

(CI: 0.27–7.16) and 0.85 (CI: 0.28–2.58) was calculated for studies with 6 days and 7 days’ intervals between discontinuation of anti-thyroid drugs and radioactive treatment, respectively. The combined effect size for subgroup analysis was 1.38 (CI: 1.07–1.79) (Table 3).

**Table 3.** Subgroup analysis based on interval between discontinuation of methimazole and initiation of radioactive iodine

	Study name/Subgroup name	Risk ratio	CI lower limit	CI upper limit	Weight	Q	p_Q_	I^2^
1	Andrade et al.^22^	1.38	0.40	4.77	66.25%			
2	Andrade et al.^20^	1.83	0.32	10.45	33.75%			
**3**	**4 days**	**1.52**	**0.28**	**8.18**	**45.01%**	**0.07**	**0.793**	**0.00%**
4	Braga et al.^21^	1.13	0.07	18.34	28.17%			
5	Burch et al.^23^	1.50	0.26	8.50	71.83%			
**6**	**6 days**	**1.38**	**0.27**	**7.16**	**46.69%**	**0.03**	**0.859**	**0.00%**
7	Pirnat et al.^24^ (12 months)	1.18	0.17	8.25	11.87%			
8	Pirnat et al.^24^ (6 months)	2.36	0.61	9.09	22.70%			
9	Pirnat et al.^24^ (3 months)	0.51	0.14	1.88	23.73%			
10	Pirnat et al.^24^ (1 month)	0.59	0.24	1.47	41.71%			
**11**	**7 days**	**0.85**	**0.28**	**2.58**	**8.30%**	**3.59**	**0.309**	**16.46%**
**12**	**Combined effect size**	**1.38**	**1.07**	**1.79**		**4.96**	**0.665**	**0.00%**

CI = confidence interval.

## DISCUSSION

Our meta-analysis showed no difference between the risk of persistent hyperthyroidism when radioactive iodine was used in conjunction with methimazole and when radioactive iodine was used alone (RR: 1.02, 95% CI: 0.62–1.66; P = 0.95, I^2^ = 0%). Subgroup analysis based on the duration between discontinuation of methimazole and the administration of radioactive iodine showed a lower risk of persistent hyperthyroidism when methimazole was discontinued within 7 days before radioactive iodine use, although this did not reach statistical significance (RR: 0.85, CI: 0.28–2.58)

Over the years, there have been debates on the use of anti-thyroid drugs in conjunction with radioactive iodine therapy for the management of hyperthyroidism in Graves’ disease, with the increasing popularity of adjunctive anti-thyroid medications with radioactive iodine therapy.^5^ To the best of our knowledge, this is the first meta-analysis to evaluate the efficacy of treatment with methimazole before the administration of radioactive iodine therapy inpatients with Graves’ disease.

In our study, we focused on the risk of persistent hyperthyroidism (treatment failure) following adjunctive treatment for Graves’ disease; our analysis showed that the risk was not significant and was only 1.02 times higher in patients treated with methimazole before radioactive iodine as compared with those who received radio-iodine therapy alone.

A similar meta-analysis done by Walter et al.^31^ on a per protocol basis revealed a summary RR of 1.34 (0.96–1.88; P = 0.09) for treatment failure with adjunctive anti-thyroid drugs compared with the control. This study differs from ours in that the authors evaluated the combined effect of adjunctive anti-thyroid drugs administered before and after radioactive iodine. In addition, some studies included in this meta-analysis had patient populations other than those with Graves’ disease.

While some studies concluded that treatment with anti-thyroid drugs during the peri-therapeutic period in patients treated with^131^I reduced the effectiveness of radioiodine, thus leading to higher treatment failure rates,^18,32,33,34,35,36,37^ we can argue that the flawed methodology and selection bias of some observational studies could have imposed some limitations.

Crooks et al.^37^ opined that methimazole has radioprotectant properties even if discontinued 6 days before administration of radioactive iodine and that the single dose RAI treatment failure rate was significantly higher in the group pretreated with methimazole (71%) than in those receiving RAI alone (25%). This correlates with the study by Connell et al.^19^ which reported a higher incidence of persistent hyperthyroidism in the group administered adjunct treatment with carbimazole 46% versus 16% (P < 0.05). One year after treatment, a similar proportion of each group had persistent thyrotoxicosis (23% in the pretreated group versus 21% in the non-pretreated group).

In a retrospective study by Tuttle et al.,^32^ pretreatment with propylthiouracil was also associated with higher treatment failure rates. Persistent hyperthyroidism was observed in 4% of patients (2/48) treated with only RAI and in 34% of patients (13/38) receiving RAI after pretreatment with propylthiouracil (P = 0.003). Patients were treated with propylthiouracil for a mean of 151 ± 32 days.^32^ Another retrospective study conducted by the authors on a later date showed that discontinuation of the anti-thyroid drug at least a week before radioactive iodine was associated with higher failure rates.^33^ The effects of propylthiouracil and methimazole/carbimazole may not be directly comparable, making it difficult to extrapolate findings from different sources that utilize varying anti-thyroid medications for adjunctive treatment.

Sabri et al.^26^ conducted a prospective randomized clinical trial that showed significantly greater success in the group without carbimazole during radioactive therapy(93% versus 49%, respectively). Stepwise logistic regression demonstrated that the failure was related to the administration of carbimazole during^131^I therapy (P < 0.005) and the absorbed dose of radioiodine (P < 0.025). It is interesting to note that in this study, simultaneous administration of the anti-thyroid drug was the decisive factor for successful radioactive iodine therapy, as16 patients who discontinued ATD 1-3 days before radioiodine therapy showed a 94% success rate. Thus, the authors recommended that, if clinically feasible, ATDs should be discontinued at least a day before the initiation of radioiodine treatment.

This is in tandem with the study by Bonnema et al.^14^ which assessed cure rates in a group receiving continuous methimazole therapy during and 4 weeks after radioactive iodine therapy versus a group that discontinued methimazole 8 days before radioiodine therapy. Patients receiving continuous methimazole had a lower cure rate (44%) than those who discontinued methimazole 8 days before radioactive iodine therapy (61%). Pirnat et al.^24^ also reported similar lower cure rates in patients who were continuously administered methimazole until radioiodine application.

Some studies have advocated adjunctive treatment with ATDs in conjunction with radioactive iodine. Kung et al.^38^ studied the use of a block replacement regimen of methimazole plus L-thyroxine on the result of radioactive iodine therapy and determined that persistent hyperthyroidism was found in 38.7% of the patients pretreated with methimazole plus L-thyroxine versus 44.5% of those who were administered radioactive iodine only. In addition, the time to achieve euthyroidism was earlier with adjunctive treatment (two versus eight weeks).^38^

Similar effectiveness and cure rates were observed in patients pretreated with methimazole compared to non-pretreated patients in two of the studies included in our meta-analysis.^21,24^ However, Burch et al.^23^ had the opinion that most patients with Graves’ disease should not be pretreated with anti-thyroid drugs before receiving radioiodine, as pretreatment with methimazole results in a rapid increase in thyroid hormone levels upon discontinuation of these medications in preparation for radioiodine therapy. This was also supported by an earlier clinical trial by Andradeet al.^20^which observed that interruption of anti-thyroid drugs caused a short-term increase in serum thyroid hormone levels in patients with Graves’ hyperthyroidism receiving radioactive iodine therapy. One year later, the pretreated group and those who received radioactive iodine therapy alone were similar in terms of persistent hyperthyroidism (15.6% in the radioactive iodine group versus 13.8% in the adjunctive methimazole group).

The 2016 American Thyroid Association guidelines state that pretreatment with methimazole before radioactive iodine therapy for Graves’ disease should be considered in patients at increased risk of complications due to worsening hyperthyroidism, and methimazole should be discontinued 2-3 days before radioactive iodine therapy.^5^ The subgroup analysis performed in our study with regard to the interval between stopping ATD and RAI therapy showed a lower risk ratio with increasing duration of discontinuation of ATD. The risk ratio was 1.52 (0.28–8.18) for a 4-day interval, 1.38 (0.27–7.16) for a 6-day interval, and 0.85 (0.28–2.58) for a 7-day interval between discontinuation of methimazole and administration of radioiodine. This implies that the risk of persistent hyperthyroidism is reduced by 15% if pretreatment with methimazole is administered 7 days before radioiodine therapy, although the observation was not considered statistically significant.

Publication bias was minimal, as the funnel plot displayed an asymmetric distribution (Egger’s t-test = 1.31, P = 0.238). There are significant side-effect profiles of ATDs, with 13% of patients developing an adverse reaction, as shown in a network meta-analysis conducted by Sundareshet al.^39^ Liver injury and elevated transaminases (worse with propylthiouracil), and dermatologic reactions are common adverse effects.^3,40^ Therefore, the choice of therapy in Graves’ disease is influenced by many factors and must be tailored to each patient’s characteristics and needs.^41,42^ Pretreatment may be considered in patients who require rapid biochemical control and are at increased risk of thyrotoxic complications.^43^

### Limitations and recommendations

The narrow eligibility criteria of our systematic review resulted in a small sample size and limited number of studies included in the final synthesis. This might have reduced the power of the study and accounted for the statistical insignificance of the results obtained. The limited number of randomized controlled trials motivates the essence of conducting well-designed RCTs with a homogenous disease population (Graves’ disease) and a larger sample size to expand the evidence needed to make informed decisions. The last randomized study reported on this subject was conducted by Pirnat et al.^24^ over nine years ago.

The only anti-thyroid drug considered in the included trials for our systematic review was methimazole. This might limit the translation of our ﬁndings with regard to other anti-thyroid medications, such as carbimazole (a precursor of methimazole) and propylthiouracil. Overall, our study showed minimal statistical heterogeneity (I^2^ = 0%) for the main outcome measure. We also included a uniform sample population with Graves’ disease only and ATD (methimazole) utilized. However, the dose of radioactive iodine utilized (fixed or adapted dose)and the varying duration of follow-up for the different studies included could be imminent sources of heterogeneity. Future systematic reviews with a subgroup analysis evaluating the varying duration of follow-up as it pertains to persistent hyperthyroidism should be conducted.

## CONCLUSIONS

This study shows that treating patients with an anti-thyroid medication(methimazole)before utilizing radioactive iodine has the same treatment failure risk as using radioactive iodine therapy alone. The use of methimazole before radioactive iodine administration was not associated with an increased risk of persistent hyperthyroidism.

Although the treatment failure risks were similar between the two groups, only the subgroup that discontinued methimazole seven days before the use of radioactive iodine had a lower risk of persistent hyperthyroidism. Concerns about medication toxicity and adverse effects should be considered when clinicians make decisions on combining therapies for the treatment of Graves’ disease.

### Key implications

- Clinicians who schedule radioactive iodine therapy for Graves’ disease treatment may not need to administer an initial methimazole use to patients, except in cases of increased risk of thyrotoxic complications, such as liver injury and dermatological reactions, which are associated with methimazole use.
- Research institutions should conduct randomized controlled trials with larger patient cohorts on treatment options for Graves’ disease to obtain statistically significant results that aid clinical decisions and improve patient outcomes.

## References

[1] Davies TF, Ando T, Lin RY, Tomer Y, Latif R (2005). Thyrotropin receptor-associated diseases: from adenomata to
Graves disease. J Clin Invest..

[2] Prabhakar BS, Bahn RS, Smith TJ (2003). Current perspective on the pathogenesis of Graves’ disease and
ophthalmopathy. Endocr Rev..

[3] Sundaresh V, Brito JP, Wang Z (2013). Comparative effectiveness of therapies for Graves’
hyperthyroidism: a systematic review and network
meta-analysis. J Clin Endocrinol Metab..

[4] Parle JV, Maisonneuve P, Sheppard MC, Boyle P, Franklyn JA (2001). Prediction of all-cause and cardiovascular mortality in elderly
people from one low serum thyrotropin result: a 10-year cohort
study. Lancet..

[5] Ross DS, Burch HB, Cooper DS (2016). 2016 American Thyroid Association Guidelines for Diagnosis and
Management of Hyperthyroidism and Other Causes of
Thyrotoxicosis. Thyroid..

[6] Furszyfer J, Kurland LT, McConahey WM, Elveback LR (1970). Graves’ disease in Olmsted County, Minnesota, 1935 through
1967. Mayo Clin Proc..

[7] Singer PA, Cooper DS, Levy EG (1995). Treatment guidelines for patients with hyperthyroidism and
hypothyroidism. Standards of Care Committee, American Thyroid
Association. JAMA..

[8] Burch HB, Burman KD, Cooper DS (2012). A 2011 survey of clinical practice patterns in the management of
Graves’ disease. J Clin Endocrinol Metab..

[9] Hamilton HB, Werner SC (1952). The effect of sodium iodide, 6-propylthiouracil, and
1-methyl-2-mercaptoimidazole during radioiodine therapy of
hyperthyroidism. J Clin Endocrinol Metab..

[10] Cooper DS (2005). Antithyroid drugs. N Engl J Med..

[11] Kahaly GJ, Bartalena L, Hegedüs L (2018). 2018 European Thyroid Association Guideline for the Management of
Graves’ Hyperthyroidism. Eur Thyroid J..

[12] Brito JP, Payne S, Singh Ospina N (2020). Patterns of Use, Efficacy, and Safety of Treatment Options for
Patients with Graves’ Disease: A Nationwide Population-Based
Study. Thyroid..

[13] Negro R, Attanasio R, Grimaldi F (2016). A 2015 Italian Survey of Clinical Practice Patterns in the
Management of Graves’ Disease: Comparison with European and North American
Surveys. Eur Thyroid J..

[14] Bonnema SJ, Bennedbaek FN, Veje A, Marving J, Hegedüs L (2006). Continuous methimazole therapy and its effect on the cure rate of
hyperthyroidism using radioactive iodine: an evaluation by a randomized
trial. J Clin Endocrinol Metab..

[15] McDermott MT, Kidd GS, Dodson LE, Hofeldt FD (1983). Radioiodine-induced thyroid storm. Case report and literature
review. Am J Med..

[16] Hayek A (1978). Thyroid storm following radioiodine for
thyrotoxicosis. J Pediatr..

[17] Sheeler LR, Skillern PG, Schumacher OP, Eversman JJ (1984). Radioiodine-induced thyroid storm: a point of
controversy. Am J Med..

[18] Reynolds LR, Kotchen TA (1979). Antithyroid drugs and radioactive iodine. Fifteen years of
experience with Graves’ disease. Arch Intern Med..

[19] Connell JM, Hilditch TE, McCruden DC, Robertson J, Alexander WD (1984). Effect of pretreatment with carbimazole on early outcome
following radio-iodine (131I) therapy. Eur J Nucl Med..

[20] Andrade VA, Gross JL, Maia AL (1999). Effect of methimazole pretreatment on serum thyroid hormone
levels after radioactive treatment in Graves’
hyperthyroidism. J Clin Endocrinol Metab..

[21] Braga M, Walpert N, Burch HB, Solomon BL, Cooper DS (2002). The effect of methimazole on cure rates after radioiodine
treatment for Graves’ hyperthyroidism: a randomized clinical
trial. Thyroid..

[22] Andrade VA, Gross JL, Maia AL (2001). The effect of methimazole pretreatment on the efficacy of
radioactive iodine therapy in Graves’ hyperthyroidism: one-year follow-up of
a prospective, randomized study. J Clin Endocrinol Metab..

[23] Burch HB, Solomon BL, Cooper DS (2001). The effect of antithyroid drug pretreatment on acute changes in
thyroid hormone levels after (131)I ablation for Graves’
disease. J Clin Endocrinol Metab..

[24] Pirnat E, Zaletel K, Gaberšček S, Hojker S (2011). The outcome of 131I treatment in Graves’ patients pretreated or
not with methimazole. Hell J Nucl Med..

[25] Karyampudi A, Hamide A, Halanaik D, Sahoo JP, Kamalanathan S (2014). Radioiodine therapy in patients with Graves’ disease and the
effects of prior carbimazole therapy. Indian J Endocrinol Metab..

[26] Sabri O, Zimny M, Schulz G (1999). Success rate of radioiodine therapy in Graves’ disease: the
influence of thyrostatic medication. J Clin Endocrinol Metab..

[27] Azizi F, Yousefi V, Bahrainian A (2012). Long-term continuous methimazole or radioiodine treatment for
hyperthyroidism. Arch Iran Med..

[28] Kung AW, Yau CC, Cheng A (1994). The incidence of ophthalmopathy after radioiodine therapy for
Graves’ disease: prognostic factors and the role of
methimazole. J Clin Endocrinol Metab..

[29] Walter MA, Christ-Crain M, Schindler C, Müller-Brand J, Müller B (2006). Outcome of radioiodine therapy without, on or 3 days off
carbimazole: a prospective interventional three-group
comparison. Eur j Nucl Med Mol Imaging..

[30] Moher D, Liberati A, Tetzlaff J, Altman DG, PRISMA Group (2009). Preferred Reporting Items for Systematic Reviews and
Meta-Analyses: The PRISMA Statement. BMJ..

[31] Walter MA, Briel M, Christ-Crain M (2007). Effects of antithyroid drugs on radioiodine treatment: systematic
review and meta-analysis of randomised controlled trials. BMJ..

[32] Tuttle RM, Patience T, Budd S (1995). Treatment with propylthiouracil before radioactive iodine therapy
is associated with a higher treatment failure rate than therapy with
radioactive iodine alone in Graves’ disease. Thyroid..

[33] Hancock LD, Tuttle RM, LeMar H, Bauman J, Patience T (1997). The effect of propylthiouracil on subsequent radioactive iodine
therapy in Graves’ disease. Clin Endocrinol (Oxf)..

[34] Körber C, Schneider P, Körber-Hafner N, Hänscheid H, Reiners C (2001). Antithyroid drugs as a factor influencing the outcome of
radioiodine therapy in Graves’ disease and toxic nodular
goitre?. Eur J Nucl Med..

[35] Oszukowska L, Knapska-Kucharska M, Makarewicz J, Lewiński A (2010). The influence of thiamazole, lithium carbonate, or prednisone
administration on the efficacy of radioiodine treatment ((131)I) in
hyperthyroid patients. Endokrynol Pol..

[36] Marcocci C, Gianchecchi D, Masini I (1990). A reappraisal of the role of methimazole and other factors on the
efficacy and outcome of radioiodine therapy of Graves’
hyperthyroidism. J Endocrinol Invest..

[37] Crooks J, Buchanan WW, Wayne EJ, Macdonald E (1960). Effect of pretreatment with methylthiouracil on results of I-131
therapy. Br Med J..

[38] Kung AW, Yau CC, Cheng AC (1995). The action of methimazole and L-thyroxine in radioiodine therapy:
a prospective study on the incidence of hypothyroidism. Thyroid..

[39] Sundaresh V, Brito JP, Wang Z (2013). Comparative effectiveness of therapies for Graves’
hyperthyroidism: a systematic review and network
meta-analysis. J Clin Endocrinol Metab..

[40] Yu W, Wu N, Li L (2020). Side Effects of PTU and MMI in the Treatment of Hyperthyroidism:
A Systematic Review and Meta-Analysis. Endocr Pract..

[41] Streetman DD, Khanderia U (2003). Diagnosis and treatment of Graves disease. Ann Pharmacother..

[42] Burch HB, Cooper DS (2015). Management of Graves Disease: A Review. JAMA..

[43] Bahn Chair RS, Burch HB, Cooper DS (2011). Hyperthyroidism and other causes of thyrotoxicosis: management
guidelines of the American Thyroid Association and American Association of
Clinical Endocrinologists. Thyroid..

